# The Effect of the Gelatinous Lactulose for Postoperative Bowel Movement in the Patients Undergoing Cesarean Section

**DOI:** 10.1155/2014/752862

**Published:** 2014-10-29

**Authors:** Daisuke Shigemi, Kazuho Nakanishi, Miwa Miyazaki, Yoshie Shibata, Shunji Suzuki

**Affiliations:** Department of Obstetrics and Gynecology, Japanese Red Cross Katsushika Maternity Hospital, Japan

## Abstract

Lactulose is a non-digestible disaccharide formed from fructose and galactose. The objective of this study was to assess the effect of lactulose on gastrointestinal function after cesarean section. One hundred patients who underwent cesarean section at the Japanese Red Cross Katsushika Maternity Hospital were enrolled in this study. They were divided into 2 groups by randomization: (1) an L group that was treated with gelatinous lactulose (*N* = 48) and (2) a control group (C group) that did not receive gelatinous lactulose (*N* = 52). The interval between cesarean section and first postoperative flatus, defecation, and walking; appearance of symptoms of ileus; use of other medicines for stimulating bowel movement; properties and state of feces; and duration of postoperative hospital stay were compared between the two groups. The two groups did not show a significant difference in postoperative outcomes, except for the incidence of loose or watery stools (50% in the L group and 26.9% in the C group, *P* = 0.03). This study could not demonstrate the apparent effectiveness of lactulose in improving bowel function after cesarean section. Therefore, a routine use of gelatinous lactulose after surgery may negatively impact the patients undergoing cesarean section.

## 1. Introduction

Delayed improvement of bowel function after surgery, including postoperative ileus, can lead to decreased mobility and postponed oral feeding, resulting in prolonged periods of hospitalization and increased healthcare costs [1]. Particularly, in women undergoing cesarean section (CS), decreased mobility may increase the risk of deep vein thrombosis or embolism, because they are at a high risk of developing thrombosis. Therefore, maintenance of normal digestive function is an essential factor affecting the recovery of postoperative paralytic ileus.

Lactulose is a nondigestible disaccharide formed from fructose and galactose. According to some recent reports [2–6], lactulose is commonly used for the treatment of chronic pediatric constipation and hepatic encephalopathy. Some Japanese studies have reported that powdered or liquid lactulose is effective in aiding postoperative gastrointestinal motor activity in obstetric and gynecologic surgeries, including total abdominal hysterectomy, myomectomy, ovarian cystectomy, and CS [7, 8]. However, there is no previous report addressing the utility of lactulose in patients after only CS. Thus, the objective of this study was to assess the effect of lactulose on gastrointestinal function after CS.

## 2. Materials and Methods

The Committee on Ethical Practice of the Japanese Red Cross Katsushika Maternity Hospital approved the study protocol and oral informed consent was obtained from all patients enrolled in this study.

**Figure 1:**
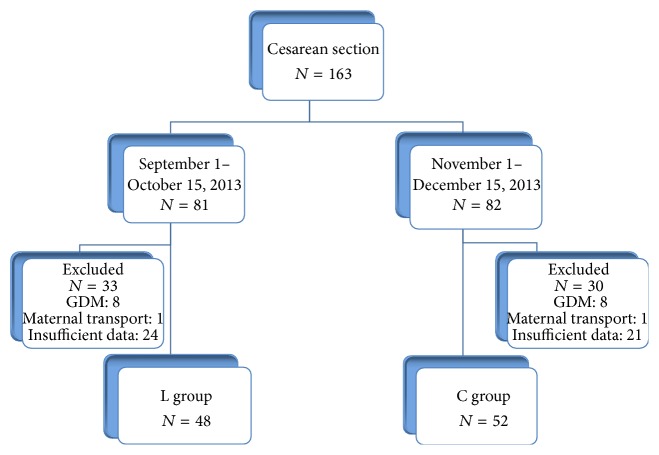
Study flow diagram.

One hundred patients who underwent CS at the Japanese Red Cross Katsushika Maternity Hospital from September 2013 to December 2013 were enrolled in this study. The patients were divided into 2 groups by randomization: (1) an L group that was treated with gelatinous lactulose (*N* = 48) and (2) a control group (C group) that did not receive gelatinous lactulose (*N* = 52) (Figure 1). Caloryl Jelly 40.496% (Sato Pharmaceutical Co., Ltd., Tokyo, Japan), containing 6.500 g of lactulose in a total amount of 16.05 g per piece, was used as the gelatinous lactulose medicine in this study. Patients with gestational diabetes mellitus, overt diabetes, severe complications of major organs, and galactosemia were excluded from the study.

Patients in the L group were prescribed 64.2 g/day of lactulose twice daily immediately after meals from the first to the third postoperative day. Patients in the C group were prohibited from being administered other bowel movement-stimulating medicines including enema, except for patients with symptoms of postoperative ileus. Patients in both groups were allowed to drink Japanese tea in the morning and have liquid food, rice porridge, and normal meal at the midday of the first postoperative day, night of the first postoperative day, and midday of the second postoperative day, respectively.

The interval between CS and first postoperative flatus, defecation, and walking; appearance of nausea, vomiting, or abdominal pain with abdominal distension as symptoms of ileus; use of other medicines for stimulating bowel movement; properties and state of feces; and duration of postoperative hospital stay were recorded for patients in both groups.

The Japanese Red Cross Katsushika Maternity Hospital had about 1800 deliveries during the past year. Approximately, 30% of patients underwent CS.

With respect to our surgical method for CS, vertical midline incision or Pfannenstiel transverse incision (approximately 3 fingerbreadths above and parallel to the symphysis pubis) was selected mainly on the basis of the patient's request in cases of a first nonurgent CS. In these cases, the bladder flap and peritoneum were attached with a running suture using polyglactin. In cases of Pfannenstiel transverse incision, the rectus fascia was also incised transversely. In this study, a transverse incision of the lower uterine segment was performed for all patients and barrier agents were used for preventing adhesions during CS.

The combined spinal epidural anesthesia (CSEA) is used for almost scheduled CS by anesthesiologists and only spinal anesthesia is usually used for emergent CS by obstetricians in our hospital. There was no case using general anesthesia in the periods of this study. The epidural catheter is extracted at midday of second postoperative day. As pain control, all patients used nonsteroidal anti-inflammatory drug (NSAID) after the end of epidural anesthesia. Although some patients had complicated mild bronchial asthma, they had used NSAID before CS and no adverse effects occurred with postoperative use of NSAID.

The results are expressed as number (%) or mean ± standard deviation. The statistical difference was determined by two-sided Student's* t*-test, chi-square test, and Yates chi-square test. Differences with *P* < 0.05 were considered significant.

## 3. Results

Table 1 shows patient demographics and operation characteristics. There were no significant differences between the 2 groups with respect to age, body mass index, numbers of past surgeries, numbers of urgent surgeries, and surgical time (Table 1).  Table 2 shows the postoperative outcomes. No significant differences were observed between the groups, except for the incidence of loose or watery stools (50% in the L group and 26.9% in the C group, *P* = 0.03). Three patients (6.3%) stopped taking lactulose because of uncomfortable loose stools and/or diarrhea (Table 2). No patients had electrolytic abnormalities which could affect bowel function.

## 4. Discussion

To the best of our knowledge, this is the first report concerning the effectiveness of gelatinous lactulose on gastrointestinal function after CS. Lactulose is not hydrolyzed by intestinal disaccharidase or absorbed in the gut; it is converted mainly to lactic and acetic acids by various bacteria in the colon. In addition to the increased osmotic effect, the lower pH in the proximal colon may reduce stool pH and lead to the stimulation of colonic propulsion [9].

Previous reports have shown that lactulose is significantly effective in chronic pediatric constipation [2, 3]. Further, lactulose can decrease the incidence of hyperammonemia and minimal hepatic encephalopathy in patients with cirrhosis or following hepatic surgery because of the reduction in colonic pH and modification of the microflora in the colon [4–6]. However, no prior study has identified the effects of lactulose on postoperative bowel function in patients after CS.

In this study, we could not show any apparent effect of gelatinous lactulose on postoperative bowel function after CS; these results are contrary to those reported by some previous studies [9, 10]. Although the mechanisms leading to our results remain unclear, several factors can be considered as possible causes. First, compared with gastric or bowel surgeries, gynecological surgery is less invasive for the intestine because of the short surgical time, less manipulation of the gut, and rare preoperative inflammation of the colonic area [10]. CS can be considered to have the same effect on gut damage as other gynecological surgeries. Second, systemic anesthesia was not performed in this study; only spinal and/or epidural anesthesia without opioids was performed. Systemic or epidural opioid anesthesia is usually more invasive for the patient's gastrointestinal motility than regional anesthesia without opioids [11]. Indeed, the appearances of nausea, vomit, or abdominal pain with abdominal distension as symptoms of ileus in this study were less than the general incidence of ileus after intra-abdominal surgeries reported by prior study [12]. Thus, there may be little need for preventive administration of oral lactulose in women who have undergone CS that induces lower damage on gastrointestinal motor function.

The number of patients in this study may have been too small to obtain any significant result. It is estimated that a definitive trial powered to detect a 60% reduction in the appearance of the symptoms of ileus would require approximately 400 patients equally divided in the control and intervention group (two-tailed *α* = 0.05, *β* = 0.2).

Although apparent effectiveness could not be detected, oral gelatinous lactulose has some merits. It can be easily used for immobile postoperative patients because of its noninvasive nature. In addition, no severe adverse event, excluding mild diarrhea, has been reported for gelatinous lactulose. Other medicines that stimulate bowel movement, such as sympathetic blocking agents and prostaglandin *F*_2_ *α*, are inconvenient immediately after surgery because they induce side effects that affect first postoperative walking, such as bradycardia and bronchial asthma, respectively. In this study, 3 patients (6.3%) stopped using lactulose because of uncomfortable loose stools or diarrhea. These were considered as mild problems with regard to the general condition of the patients because they lasted only few days and did not result in an electrolytic abnormality.

## 5. Conclusion

Our study could not demonstrate any apparent effectiveness of lactulose in improving bowel function in patients after CS. Therefore, a routine use of gelatinous lactulose after surgery may negatively impact the patients undergoing cesarean section. However, considering the recent increase in the number of CSs, a further study may be required.

## Figures and Tables

**Table 1:** Patient demographics and operation characteristics.

	L group	C group	
	(*N* = 48)	(*N* = 52)	
Maternal age (years)	33 ± 5.61	34.35 ± 4.89	*P* = 0.20
Numbers of previous deliveries	0.71 ± 0.80	0.69 ± 0.78	*P* = 0.92
BMI (kg/m^2^)	25.85 ± 4.04	25.95 ± 3.77	*P* = 0.90
Delivery age (weeks)	37.04 ± 2.16	36.79 ± 3.05	*P* = 0.64
Past history			
Previous CSs	18 (37.5%)	21 (40.4%)	*P* = 0.77
Previous abdominal surgeries except CS	9 (18.8%)	6 (11.5%)	*P* = 0.31
Urgent CS	19 (39.6%)	18 (34.6%)	*P* = 0.61
Natural pregnancy	38 (79.2%)	45 (86.5%)	*P* = 0.33
Anesthesia			
Epidural and spinal	35 (72.9%)	39 (75%)	*P* = 0.81
Spinal only	13 (27.1%)	13 (25%)	*P* = 0.81
Skin vertical incision	37 (77.1%)	38 (73.1%)	*P* = 0.64
Surgical time (min)	32.31 ± 7.64	30.96 ± 7.57	*P* = 0.38
Intraoperative bleeding (mL)	721.29 ± 601.56	789.52 ± 411.78	*P* = 0.51
Blood transfusion	2 (4.2%)	3 (5.8%)	*P* = 0.93
Meconium stain of the amniotic fluid	4 (8.3%)	2 (3.8%)	*P* = 0.60
Intra-abdominal adhesion	4 (8.3%)	6 (11.5%)	*P* = 0.59

BMI: body mass index; CS: cesarean section.

Data are presented as number (%) or mean ± standard deviation.

**Table 2:** Postoperative outcomes.

	L group	C group	
	(*N* = 48)	(*N* = 52)	
Time interval between CS and the following:			
first flatus (h)	25.26 ± 15.57	28.80 ± 16.98	*P* = 0.29
first defecation (h)	59.73 ± 27.37	65.6 ± 25.1	*P* = 0.28
first walking (h)	25.21 ± 7.90	24.45 ± 7.56	*P* = 0.63
Appearance of nausea, vomiting, or abdominal pain with abdominal distension	3 (6.25%)	8 (15.4%)	*P* = 0.25
Use of other medicines for stimulating bowel movement	5 (10.4%)	9 (17.3%)	*P* = 0.48
Loose stools or diarrhea	24 (50%)	14 (26.9%)	*P* = 0.03
Duration of postoperative hospital stay (days)	7.33 ± 0.63	7.44 ± 0.61	*P* = 0.43

CS: cesarean section.

Data are presented as number (%) or mean ± standard deviation.
